# Supplementary material for: Impact of Polyvascular Disease on Long-Term Prognosis of Patients with Acute Coronary Syndrome—A Retrospective Cohort Study in Italy
Source: J Clin Med. 2025 Jun 11;14(12):4158. doi: 10.3390/jcm14124158 (PMC12194242; doi:10.3390/jcm14124158)
Supplement: Supplementary file 1 [file jcm-14-04158-s001.zip › jcm-3648971-supplementary.pdf]

Supplementary material

Impact of Polyvascular Disease on Long-Term Prognosis of Patients with Acute Coronary Syndrome—A Retrospective Cohort Study in Italy

Gian Francesco Mureddu, Stefano Rosato, Paola D'Errigo, Pompilio Faggiano, Paola Ciccarelli, Gabriella Badoni, Marco Ambrosetti, Francesco Fattirolli and Giovanni Baglio

Content

Supplemental Table S1. ICD9-CM codes used to retrieve information on exposure categories (a) and risk factors and comorbidities (b)

Supplemental Table S2. Multivariate models for 5 years death and rehospitalization for HF, Ischaemic Stroke and AMI. Hazard Ratio (HR)/Sub-hazard Ratio (SHR) and confidence interval (CI)

Supplemental Figure S1. Kaplan-Meyer survival curves for 5 years death (A) and Cumulative Inidence Curve for 5-year re-hospitalization for AMI (B), re-hospitalization for Heart Failure (C), re-hospitalization for Stroke (D)

Supplemental Table S1. ICD9-CM codes used to retrieve information on exposure categories (a) risk factors and comorbidities (b)

	ICD9 Code	
	**Diagnosis - Index or previous admission**	**Procedures - Index or previous admission**
**a) Exposure**		
Peripheral Arterial Disease (PAD)	250.70, 250.71, 250.72, 250.73, 440.2, 440.3, 440.4, 440.8, 440.9, 443.9, 444.22, 444.81, 445.02	38.08, 38.16, 38.18, 38.38, 38.48, 38.68, 39.49, 39.50, 39.56, 39.57, 39.58, 39.90, 39.25, 39.29, 84.1, 84.91
Cerebrovascular Disease (CeVD)	433.x1, 434.x1, 436 (excluded if 430, 431, 432 are simultaneously present)	00.61, 00.62, 00.63, 38.10, 38.11, 38.12
**b) Risk factors and comorbidities**		
	**Diagnosis - Index or previous admission**	**Procedures - Index or previous admission**
Hypertension	(401-405)*	
Diabetes	(250.0-250.9 [except 250.70-250.73])*	
Heart failure	428	
Ill-defined descriptions and complications of heart disease	429*	
Chronic coronary syndromes	(411, 413, 414)*	
Arrhythmias	426, 427	
Anemia	280-284, 285 (except 285.1)	
Blood clotting defects	286	
Cardiomyopathy	425	
Rheumatic heart disease	391*, 393-398	
Endocarditis and acute myocarditis	421, 422	
Other chronic heart conditions	745, V15.1, V42.2, V43.2, V43.3, V45.0	
Chronic kidney diseases	582, 583, 585-588	
Chronic disease (liver, pancreas, intestine)	571, 572, 577.1-577.9, 555, 556	
Obesity	728.0*	
Chronic obstructive pulmonary disease	491, 492, 494, 496	
Malignant neoplasms	140.0–208.9, V10	
Coagulation disorders	286	
Previous AMI	410*, 412	
Previous CABG	V45.81	36.1*
Previous PCI	V45.82	(00.66, 36.0)*
Other previous cardiac surgery than CABG		(35, 37.0, 37.1, 37.3, 37.4, 37.5, 37.6, 37.9)*

*Retrieved only in previous admission

Supplemental Figure S1. Kaplan-Meyer survival curves for 5 years death (A) and Cumulative Inidence Curve for 5-year re-hospitalization for AMI (B), re-hospitalization for Heart Failure (C), re-hospitalization for Stroke (D)

A)

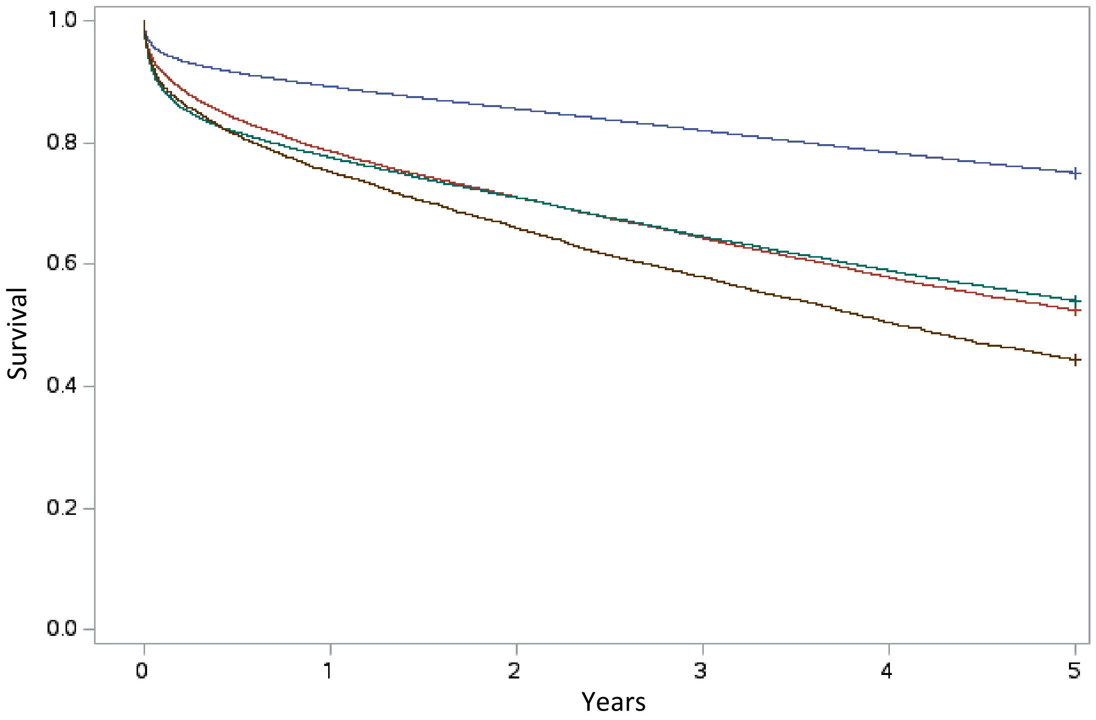

B)

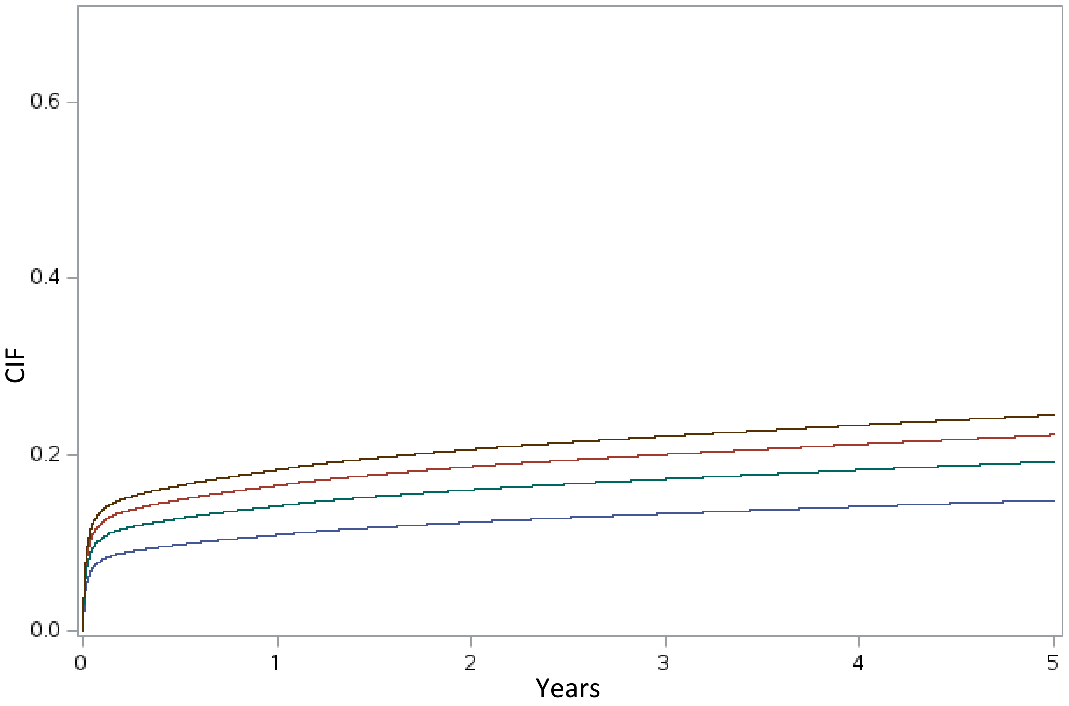

C)

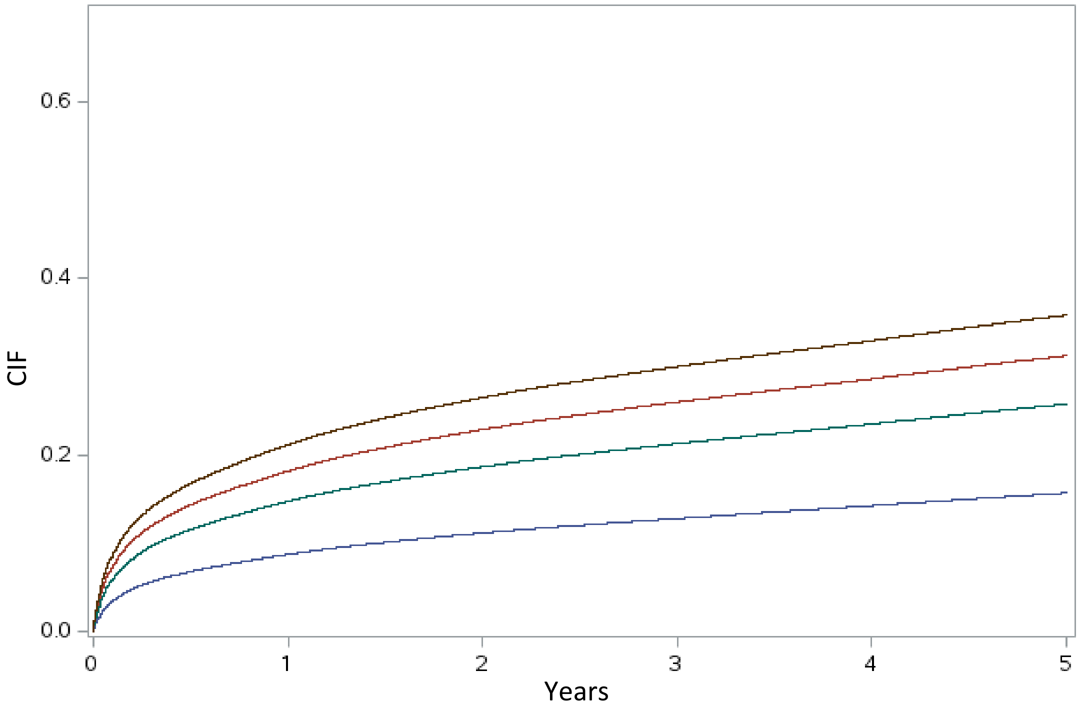
0.6
0.4
0.2
0.0
CIF
0
1
2
3
4
5
Years

D)

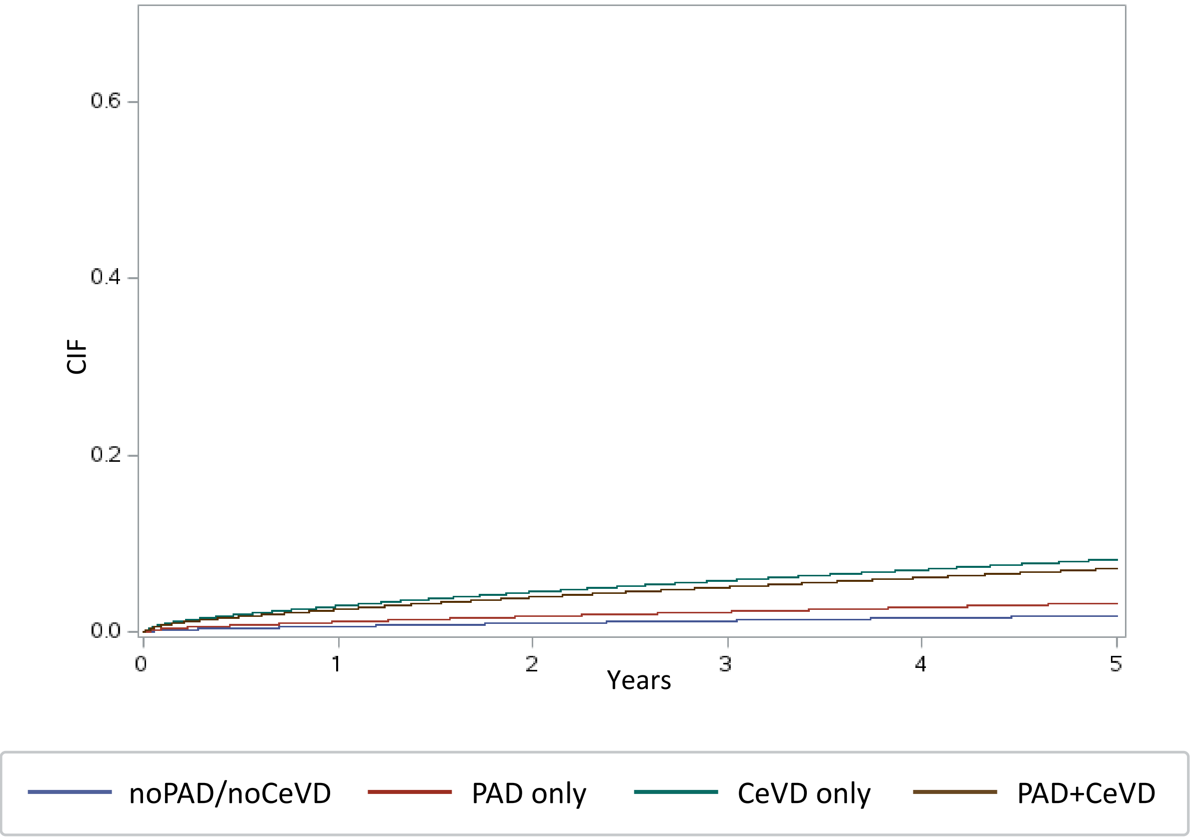
0.6
0.4
0.2
0.0
CIF
0
1
2
3
4
5
Years
noPAD/noCeVD
PAD only
CeVD only
PAD+CeVD

Supplemental Table S2. Multivariate models for 5 years death and rehospitalization for HF, Ischaemic Stroke and AMI. Hazard Ratio (HR)/Sub-hazard Ratio (SHR) and confidence interval (CI)

	Death			HF			Stroke			AMI		
	HR	95% CI		SHR	95% CI		SHR	95% CI		SHR	95% CI	
PAD only	1.48	1.45	1.51	1.321	1.285	1.358	1.54	1.43	1.67	1.27	1.24	1.31
CeVD only	1.30	1.27	1.33	1.156	1.117	1.196	3.84	3.60	4.09	1.18	1.14	1.23
PAD+CeVD	1.52	1.46	1.57	1.336	1.27	1.405	3.22	2.90	3.57	1.32	1.25	1.40
Female gender	0.93	0.91	0.94	0.96	0.942	0.978	1.03	0.99	1.08	0.96	0.94	0.98
Age	1.08	1.08	1.08	1.024	1.023	1.024	1.02	1.02	1.02	1.00	1.00	1.00
Malignant neoplasms	1.65	1.61	1.68	0.945	0.914	0.976	1.00	0.92	1.09			
Diabetes	1.28	1.26	1.31	1.14	1.112	1.17	1.22	1.14	1.30	1.11	1.08	1.14
Obesity	1.18	1.13	1.24	1.246	1.18	1.316						
Anemia	1.34	1.31	1.37	1.077	1.046	1.108				1.10	1.07	1.14
COPD	1.26	1.24	1.29	1.219	1.187	1.251				1.07	1.03	1.10
Chronic Kidney Diseases	1.47	1.45	1.50	1.247	1.218	1.276	1.00	0.94	1.07	1.22	1.19	1.25
Other chronic disease (liver, pancreas, bowel)	1.44	1.38	1.50	1.082	1.017	1.151				1.05	0.98	1.13
Coagulation disorders	1.21	1.06	1.38									
Hearth failure	1.67	1.65	1.70	3.924	3.844	4.005	1.05	0.99	1.12	1.28	1.25	1.30
Rheumatic heart disease	1.17	1.13	1.21	1.201	1.146	1.258	1.28	1.13	1.45	1.09	1.03	1.16
Cardiomyopathy	1.19	1.15	1.22	1.505	1.448	1.563	1.26	1.12	1.42			
Arrhythmias	1.32	1.31	1.34	1.25	1.226	1.274	1.07	1.01	1.12	1.01	0.99	1.03
Endocarditis and acute myocarditis	1.42	1.25	1.60				1.60	1.08	2.38			
Other chronic heart disease				1.299	1.256	1.343				1.09	1.05	1.14
Previous AMI	1.10	1.08	1.12	1.087	1.06	1.114	1.09	1.03	1.16	0.88	0.85	0.90
Previous other chronic coronary syndromes	1.11	1.09	1.13	0.962	0.939	0.986				0.74	0.72	0.76
Previous CABG	0.88	0.86	0.90	1.331	1.293	1.371				1.41	1.37	1.46
Previous PCI	0.79	0.78	0.81	1.306	1.275	1.338				2.78	2.71	2.85
Other previous cardiac surgery than CABG	1.10	1.05	1.15							0.79	0.74	0.84

Diagnosis in index admission: Other ischemic heart disease	Ref.			Ref.			Ref.			Ref.		
Angina pectoris	1.45	1.42	1.49	1.159	1.127	1.193	1.10	1.02	1.18	1.61	1.55	1.67
AMI	1.97	1.93	2.02	1.25	1.219	1.282	1.18	1.11	1.25	4.12	3.99	4.25
